# Fate of Functional Bacterial and Eukaryotic Community Regulated by Earthworms during Vermicomposting of Dewatered Sludge, Studies Based on the 16S rDNA and 18S rDNA Sequencing of Active Cells

**DOI:** 10.3390/ijerph18189713

**Published:** 2021-09-15

**Authors:** Jun Yang, Kui Huang, Lansheng Peng, Jianhui Li, Aozhan Liu

**Affiliations:** 1 School of Environmental and Municipal Engineering, Lanzhou Jiaotong University, Lanzhou 730070, China; yangjun@mail.lzjtu.cn (J.Y.); 0219140@stu.mail.lzjtu.cn (L.P.); 0618086@stu.mail.lzjtu.cn (J.L.); 0618116@stu.mail.lzjtu.cn (A.L.); 2 Key Laboratory of Yellow River Water Environment in Gansu Province, Lanzhou 730070, China

**Keywords:** vermicompost, sludge recycling, biodegradation, microbial community, propidium monoazide

## Abstract

DNA sequencing of active cells involved in vermicomposting can clarify the roles of earthworms in regulating functional microorganisms. This study aimed to investigate the effect of earthworms on functional microbial communities in sludge by comparing biodegradation treatments with and without earthworms. PCR and high throughput sequencing based on pretreatment of propidium monoazide (PMA) were used to detect the changes in active bacterial 16S rDNA and eukaryotic 18S rDNA during vermicomposting. The results showed that the nitrate in sludge vermicomposting and control were significantly different from day 10, with a more stable product at day 30 of vermicomposting. Compared with the control, the Shannon indexes of active bacteria and eukaryotes decreased by 1.9% and 31.1%, respectively, in sludge vermicompost. Moreover, Proteobacteria (36.2%), Actinobacteria (25.6%), and eukaryotic Cryptomycota (80.3%) were activated in the sludge vermicompost. In contrast, the control had Proteobacteria (44.8%), Bacteroidetes (14.2%), Cryptomycota (50.00%), and Arthropoda (36.59%). Network analysis showed that environmental factors had different correlations between active bacterial and eukaryotic community structures. This study suggests that earthworms can decrease the diversity of bacterial and eukaryotic communities, forming a specific-functional microbial community and thus accelerating organic matter decomposition during vermicomposting of dewatered sludge.

## 1. Introduction

A large amount of excess sludge is generated in wastewater treatment plants worldwide due to increased urbanization. The sludge with a water content of 80% exceeded 60 million tons in 2019 in China and could rise to 90 million tons in 2025 [1]. Relative to other biosolids, the characteristic of excess sludge is more complicated, because it receives lots of organic substances and microorganisms from primary and secondary sedimentation tanks [2]. As a result, the above status poses a great challenge to treating and disposing of excess sludge, especially for low-income countries. Among the current treatment methods, including incineration, thermal dry, clean fuel, composting, and anaerobic digestion [3], vermicomposting is an environmentally friendly and sustainable approach for sludge recycling [4,5,6]. Earthworms can decompose various excess sludges, such as primary sedimentation tank sludge [7], anaerobic digestion sewage sludge [8,9], and dewatered sludge [4,10,11,12,13]. The final sludge vermicompost contains homogeneous particle with available nitrogen, phosphorus, potassium, micro-nutrients, and diverse microbial flora [5,14]. Therefore, the sludge vermicompost can be an agricultural organic fertilizer.

Previous studies have demonstrated that vermicomposting can realize the rapid stabilization of dewatered sludge by modifying the physicochemical properties and microbial community of sludge due to the interaction of earthworms and microorganisms during the biodegradation process [6,15]. Earthworms can directly degrade organic matter through their feeding and intestinal digestion [16]. Meanwhile, earthworms as drivers can also affect microbial growth and reproduction through their burrowing, defecation, and mucus secretion, indirectly stimulating the degradation of organic matter [17]. Molecular technique development has further shown that microorganisms, such as bacteria and eukaryotes, promote the degradation of organic matter in the vermicomposting process [4,13,18,19,20]. Notably, only active microorganisms can promote metabolism, reproduction, and other activities in the biodegradation system, known as functional microbes. However, previous studies on microbial community structure in vermicomposting used the total DNA sequences based on the techniques of phospholipid fatty acids (PLFA), polymerase chain reaction, and denaturing gradient gel electrophoresis (PCR-DGGE), high-throughput sequencing, and metagenomic sequencing [10,13,21]. The nucleic acid detection based on the total DNA cannot distinguish between active and dead cells since DNA from dead cells can stay active for a long time [22], leading to the overestimation of functional microorganisms. Therefore, the active functional microbes involved in the vermicomposting process should be investigated for enhanced clarity on the mechanism of the stabilization process of sludge. However, no study has reported on the active microorganisms in vermicomposting of sludge.

PMA, a DNA embedding dye, can effectively discriminate the active and dead microorganisms. It inhibits DNA amplification in dead cells by penetrating the damaged cell membrane and interact with extracellular DNA sequences under LED light [23]. Moreover, DNA sequencing combined with PMA straining has been used to assess the active microbial community in the environment [9,24]. However, some studies have evaluated the active microbial community in the vermicomposting system using the 16S rDNA and 18S rDNA sequencing combined with propidium monoazide.

This study aimed to investigate the changes in the active microbial community during vermicomposting of dewatered sludge and to reveal the effect of earthworms on the active microbial community in the vermicomposting system. PMA staining combined with PCR and high-throughput sequencing technology was used to detect bacterial 16S rDNA and eukaryotic 18S rDNA in sludge.

## 2. Materials and Methods

### 2.1. Experimental Design

This study used young *Eisenia fetida* as the earthworm species and a plastic box (60 cm × 40 cm × 30 cm) as vermi-reactor. The dewatered sludge was obtained from the dewatering workshop of a wastewater treatment plant in Anning District of Lanzhou city, China.

To avoid the anaerobic environment, the fresh dewatered sludge broken into about 5 mm particles was used as a vermicomposting substrate, following the method of Fu [4]. The physicochemical properties of the initial sludge are shown in Table 1.

**Figure 1:**
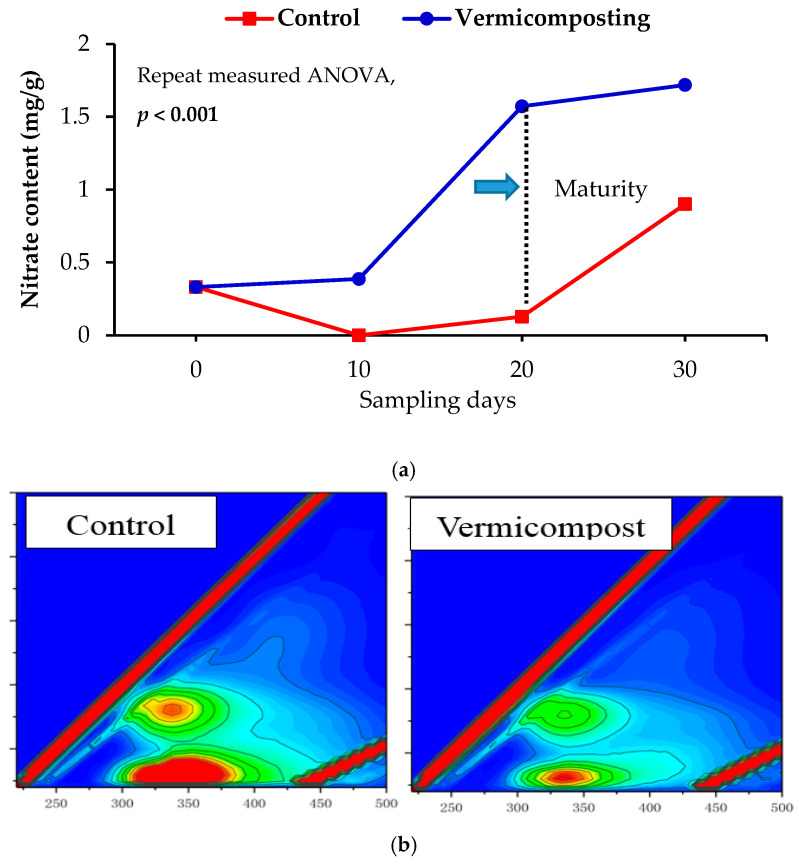
Changes in nitrate content (**a**) and dissolved organic carbon; (**b**) detected by 3D-EEM in vermicompost and control.

Vermicomposting treatment (with earthworms) and control treatment (without earthworms) were in a parallel setup. Each treatment was performed in triplicate. First, 10 kg fresh dewatered sludge was put into each vermi-reactor, then 1200 *E. fetida* were inoculated into the vermi-reactor to facilitate rapid sludge stabilization. All reactors were covered using a plastic lid to maintain a dark environment for earthworms. The vermicomposting experiment was performed at room temperature (18–28 °C). Tap water was splashed using a sprinkling can to keep a moist environment during vermicomposting. The sludge substrate was turned over once a week to maintain the oxygen content. Most sludge turned into granules, and the nitrate content significantly increased after 20 days (Figure 1). Therefore, the *E. fetida* was removed from the vermi-reactor by hand, and then their weights were measured. Subsequently, the mixture of non-digested sludge and vermicompost continued to maturate for 10 days. The fresh samples were randomly collected from the control reactor, and vermi-reactor and each was divided into two parts. One part was dried and grounded for the determination of the physical and chemical properties, and the other part was treated with the PMA dye.

### 2.2. Assay of Physicochemical Properties

The physicochemical properties were determined as described in the earlier study [12]. Briefly, the mixture of dry sample and deionized water (dry sample: water = 1:50, mass concentration) was stirred via magnetic force for 30 min, then centrifuged at 4000 rpm. The resulting upper supernatant was used for the chemical determination. A pH meter (PHS-3C, Leici, Shanghai, China) and conductivity meter (DDS-307, Leici, Shanghai, China) were used to determine the pH value and electrical conductivity (EC), respectively. After drying the sample at 105 °C for 12 h, organic matter (OM) was measured by an oven at 600 °C for 2 h. Ultraviolet spectrophotometry (HJ/T 346-2007, Chinese Standard) was used to determine nitrate-nitrogen (NO_3_^−^). A multi-parameter water quality analyzer (CNPN-7SII, Luheng, Hangzhou, China) was used to determine ammonia nitrogen (NH_4_^+^). Carbon and nitrogen analyzer (Multi N/C 2100, Jena, Germany) was used to determine dissolved organic carbon (DOC). Moreover, the DOC structure was analyzed via three-dimensional emission and excitation modes (3D-EEM) using a fluorescence spectrophotometer (RF-5300PC, SHIMADZU, Shanghai, China), as described by Huang [14].

### 2.3. PMA Treatment and DNA Extraction

Fresh sample (1 g), 200 mL DNA-free water, and 2 mL phosphate-buffered solution were fully mixed via a magnetic force for 30 min. A 20 μM PMA (5 μL) (Biotium, USA) was added into 2 mL of the mixture, then kept at 4 °C for 10 min. Subsequently, the stained sample was put in an LED lighting device (Takara, Crosslinker 12, Tokyo, Japan) for illumination (20 min). The stained sample was manually turned over every 5 min during the illumination process. DNeasy^®^ PowerSoil^®^ Kit (QIAGEN, Germany) was used to immediately extract the total genomic DNA from the treated samples (with PMA dye) following the manual instructions. The extracted DNA was checked via electrophoresis with 2% agarose gel and stored in the refrigerator at −20 °C.

### 2.4. PCR and Sequencing Methods

The V3–V4 region of 16S rDNA genes was amplified using the following primers: 341F (5′-CCTACGGGAGGCAGCAG-3′) and 806R (5′-GGACTACVSGGGTATCTAAT-3′). The 528F (5′-GCGGTAATTCCAGCTCCAA-3′)/706R (5′-AATCCRAGAATTTCACCTCT-3′) was used to amplify the V4 region of 18S rDNA genes. All primers were conjugated with barcode base pairs before amplification. All PCR reactions were conducted using the Phusion^®^ High-Fidelity PCR Master Mix (New England Biolabs). The PCR products were detected using 2% agarose gels and then purified with a GeneJET Gel Extraction Kit (Thermo Scientific, Shanghai, China). The TruSeq^®^ DNA PCR-Free Sample Preparation Kit was used to establish the sequencing library, and its quality was assessed using the Qubit (Thermo Scientific, Shanghai, China). Subsequently, the library was sequenced on NovaSeq6000 with 2 × 150 bp at the Nova biological information company (Beijing, China).

The raw reads were first filtered to obtain high-quality clean tags based on Qiime (V1.9.1) quality control processes. The tag sequences were then compared with the species annotation database using VSEARCH binaries [25]. High-quality tagged sequences were obtained after removing the chimeric sequences. Clustering of the OTUs (Operational Taxonomic Units) was set at >97% similarity using the Uparse package (Uparse v7.0.1001). A representative sequence for each OTU was taxonomically classified by comparing it with the Silva132 database [26] using Classifier (Version 11.1).

### 2.5. Statistical Analysis

The average of individual weight was calculated with difference values of final average weight of earthworms divided by their numbers minus initial average weight of earthworms divided by their numbers. All data presented are mean and standard deviations (*n* = 3). SPSS v26 software was used for one-way ANOVA analysis at 95% confidence level to evaluate the inter-group differences of physicochemical properties or sequencing results among three treatments. Excel (2016) was used to draw the charts of physicochemical properties of bacteria and eukaryotes and the histogram of α diversity and relative abundance at the gate level. Origin 2017 software was used to analyze the genus level of bacteria and eukaryotes. Gephi v0.9.2 was used to draw the correlation network analysis between bacteria and eukaryotes vs. environmental factors.

## 3. Results and Discussion

### 3.1. Maturation Assessment of Vermicomposting

The average individual weight of *E. fetida* increased from 0.39 g to 0.60 g (growth rate, 53.8%) after vermicomposting for 20 days. In addition, large amounts of cocoons and clitellate earthworms were observed in this study, suggesting that earthworms can better inhabit the sludge environment. The increased earthworm biomass indicates that the earthworms can effectively survive in the dewatered sludge, even at a higher density.

The maturation of sludge vermicompost was significantly associated with the changes in physicochemical properties of sludge during the vermicomposting process. EC, DOC, ammonium, and nitrate were used to assess the maturation of sludge vermicompost (Table 1) [10,27]. EC in each product significantly increased compared with the initial sludge (*p* < 0.05), displaying the highest value in the sludge vermicompost. EC value in vermicompost significantly increased by 1.82 times compared with that of the control group (*p* < 0.05), indicating that the activity of earthworms can effectively promote the conversion of organic matter of sludge into soluble salt [4]. Moreover, the organic matter content significantly decreased in both vermicomposting and control treatments, showing a significantly lower level in sludge vermicompost (*p* < 0.05). Alike to organic matter, the DOC content significantly decreased in vermicompost and control treatments (*p* < 0.05) (75.5% and 78.6%, respectively), after 30 days. DOC was significantly lower in vermicompost than in the control (*p* < 0.05), possibly because the organic matter in sludge was first fed on by earthworms and thus transformed into small molecular organic particles for microorganisms. Moreover, both microbial byproduct-like substances and aromatic-like substances were lower in sludge vermicompost than in control (Figure 1b), indicating that earthworms can quickly stabilize sludge, similar to previous research [11].

Nitrate content is usually used to evaluate the stability of vermicomposting products [28]. Nitrate content in vermicomposting treatment was significantly (*p* < 0.05) increased by 3.2 times from day 10 to 20 (Figure 1a). In contrast, the nitrate content in the control treatment was not changed within the first 20 days. Moreover, nitrate content was significantly different between vermicomposting and control treatments (*p* < 0.05). Nitrate content was higher in sludge vermicompost (1.91 times) than in control. These results indicate that earthworms can directly modify the population structure and abundance of ammonia-oxidizing bacteria and archaea, and thus promoting the nitrification process of sludge [29]. Ammonium is used to represent the fertility of sludge vermicompost. Herein, the ammonia nitrogen content was significantly decreased in control by 27.7% (*p* < 0.05) compared with the sludge vermicompost (Table 1). The increased ammonia nitrogen in vermicompost may be due to the direct effect of the urine, mucus, and enzymes secreted by earthworms [30]. Moreover, ammonification could have occurred in the vermicompost due to the decomposition of soluble organic matter in sludge.

Herein, the higher contents of EC, ammonium, nitrate, and lower dissolved organic carbon in vermicomposting product suggest that sludge vermicompost is completely maturated within 30 days. This rapid vermicomposting method with higher earthworm density may benefit sludge stabilization since it is less time-consuming than others in previous studies [4,13].

### 3.2. Changes in Active Bacterial and Eukaryotic Diversity

The high throughput sequencing showed that the average OTUs of bacterial 16S rDNA in initial sludge, control, and vermicompost were 1959, 1630, and 1496, respectively. Herein, fewer bacterial OTUs were associated with the inhabitation of the dead cell by PMA for DNA sample, compared with previous sequencing results [13,28]. Moreover, the average OTUs of eukaryotic 18S rDNA in initial sludge, control, and vermicompost were 182, 210, and 208, respectively. These are the highest OTUs of eukaryotic 18S rDNA recorded in sludge vermicompost to the best of our knowledge.

**Figure 2:**
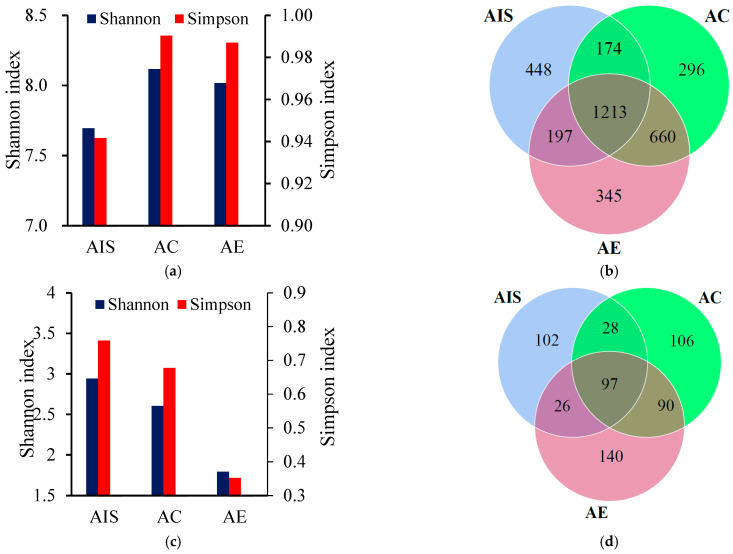
α diversity of active bacteria (**a**); eukaryotes (**c**); Venn diagram of active bacteria (**b**); eukaryotes (**d**) in initial sludge (AIS); final products of control (AC); vermicompost (AE). All data were based on OUT results.

The Shannon and Simpson indexes representing the α diversity of microorganisms are illustrated in Figure 2. The Shannon index of active bacteria in the control product and sludge vermicompost were significantly increased by 5.6% and 4.3%, respectively, compared with the initial sludge (Figure 2a) (*p* < 0.05). However, the Shannon index was not significantly different between vermicompost and control (*p* > 0.05). Similarly, the Simpson index showed a similar trend in both treatments. Earthworms can enrich the diversity of the bacterial community in vermicompost after vermicomposting, as determined using the DGGE method [4], PLFA method [31], high-throughput sequencing method [12,13], and metagenomic sequencing [14]. Herein, although the Shannon index increased after vermicomposting, earthworms did not significantly enhance the α diversity of bacteria compared with the control. This could be because this study only focused on active bacteria in vermicomposting instead of active and dead bacteria. Besides, the Venn diagram based on bacterial OUTs result (Figure 2b) identified 345 specific OTUs in sludge vermicompost (16.6% higher than in control), indicating that vermicomposting can increase some specific functional bacteria in sludge vermicompost. Earthworms can release indigenous microorganisms into the inhabited environment through casting behavior [32]. Moreover, the dissolved nutrients transformed by earthworms can enrich bacterial diversity [33].

Eukaryotes in excess sludge are mainly comprised of protists and fungi. For active eukaryotes, both Shannon and Simpson indexes showed a downward trend, with a significant decrease in sludge vermicompost compared with the initial sludge (Figure 2c). The decreased α diversity of eukaryotes in control could be due to the variations of environmental conditions in sludge during the experiment. The lower α diversity of eukaryotes in sludge vermicompost may be because earthworms feed on eukaryotes, especially the protist [34,35]. Therefore, the reduced diversity of active eukaryotes indicates that the specific and functional eukaryotic community occurred in sludge vermicompost, since effective eukaryotic tags were not significantly different between control and sludge vermicompost. In contrast, previous studies have indicated that the diversity of the eukaryotic community increases after vermicomposting of sludge based on the DGGE diagram [4] and PLFA method [31]. The difference may be because earlier studies assessed the total DNA gene in the vermicompost, which may overestimate the eukaryotes.

### 3.3. Changes in Active Bacterial and Eukaryotic Community Components

#### 3.3.1. Active Bacterial Community

**Figure 3:**
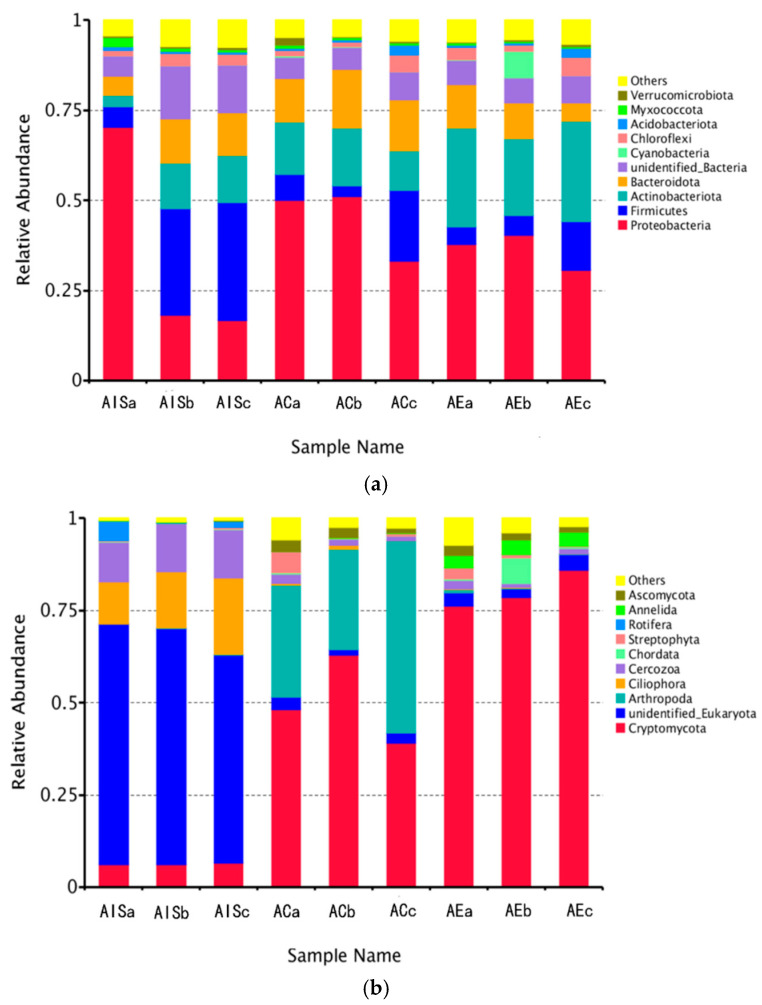
Relative abundances of active bacteria (**a**) and eukaryotes; (**b**) at phylum level in initial sludge (AIS) and final products of control (AC) and vermicompost (AE). a, b and c represent the triplicate of each sample.

Proteobacteria (17.47%), Firmicutes (31.08%), Actinobacteria (12.94%), and Bacteroidetes (12.00%) were the dominant phyla of active bacteria in initial sludge (Figure 3a). The members of Proteobacteria, Actinobacteria, and Firmicutes, as core microbiota in activated sludge in wastewater treatment systems [36], are essential in organic matter decomposition and nutrient cycling. The average abundances of active Proteobacteria and Actinobacteria in sludge vermicompost increased by 36.2% and 25.6%, respectively, compared with the raw sludge. The total abundances of Proteobacteria and Actinobacteria are the dominant phyla in many vermicomposts [13,14,33,37,38], consistent with this study. In contrast, the active abundances of Proteobacteria, Firmicutes, and Bacteroidetes in sludge vermicompost were decreased by 23.75%, 74.65%, and 24.62%, respectively, compared with the control. However, previous studies reported that the abundances of Firmicutes and Bacteroidetes significantly increased during sludge vermicomposting [12,13], which may be related to the inability to distinguish living and dead bacteria in earlier studies.

**Figure 4:**
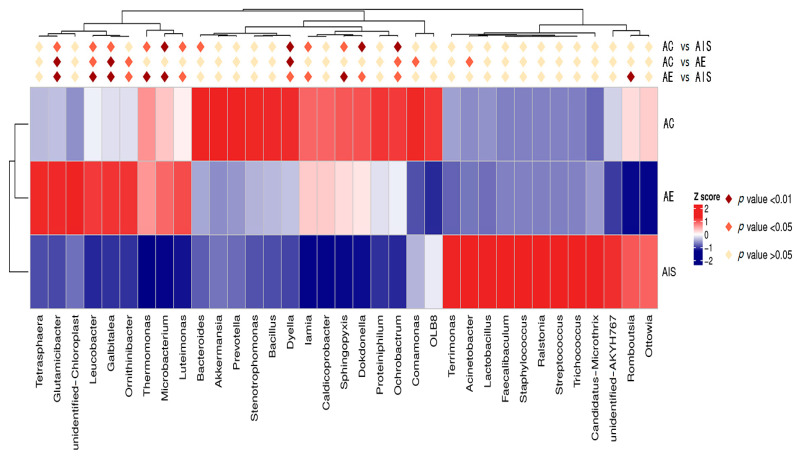
Heatmap of dominant bacterial genus and significant differences among initial sludge (AIS) and final products of control (AC) and vermicompost (AE).

Significant differences of the top 20 active bacterial genera in initial sludge, sludge vermicompost, and control are shown in Figure 4. The bacterial genus of *Candidatus microthrix*, *Ottowia*, *Acinetobacter*, and *Romboutsia* were activated in raw sludge. The dominant bacterial genus was significantly different between sludge vermicompost and control treatment (*p* < 0.05). *Dyella*, *Ochrobactrum,* and *Comamonas* were significantly dominated in the control (*p* < 0.05), while the *Glutamicibacter*, *Leucobacter*, *Calbitalea*, and *Orinithinibacter* were significantly predominated in sludge vermicompost (*p* < 0.05). These results further indicate that earthworms can significantly modify the bacterial community composition in sludge during vermicomposting. The members of *Glutamicibacter* were isolated from night-soil compost, with the features of cold-adaptation, efficient degradation, and plant growth-promotion [39]. *Leucobacter* genus is often detected in compost and the gut of soil animals, and has a denitrifying ability in the anaerobic environment [40].

#### 3.3.2. Active Eukaryotic Community

The unidentified Eukaryota (61.9%), Ciliophora (15.84%), Cercozoa (12.31%), and Cryptomycota (6.30%) were the active eukaryotes in the initial sludge (Figure 3b). Matsunaga et al. [41] indicated that the above eukaryotes can be obtained from activated sludge. The abundance of unidentified Eukaryota significantly decreased in the control product, and the members of Cryptomycota (50.00%) and Arthropoda (36.59%) become predominant. Meanwhile, the Cryptomycota (80.3%) predominated in the sludge vermicompost, followed by Annelida (3.7%), unidentified Eukaryota (3.3%), and Ascomycota (2.1%). The members of Ciliophora, Cercozoa, and Arthropoda belonging to the protist kingdom significantly decreased during vermicomposting, compared with the raw sludge, probably due to predation by earthworms. A recent study reported that the most abundant top phyla of eukaryotes in the gut of earthworms mainly belong to Cercozoa, Apicomplexa, Peronosporomycetes, Ciliophora, fungi, and nematodes [42], suggesting that earthworms can digest these eukaryotes. However, Monroy et al. [43] reported that the presence of earthworms in the soil decomposition process benefits the arthropod groups of springtails and mesostigmatid mites. Fu et al. [10] also reported that Cercozoa is dominant in the vermicomposting system using the DGGE method. The difference could be due to the aeration condition promoted by the burrowing behaviors of earthworms, which can facilitate the growth and propagation of protozoa [31]. Besides, the number of protozoa is significantly associated with the species of protozoa and earthworms herein, according to Monroy et al. [35]. The Cryptomycota, as a new cognitive phylum of fungi, are propagated in sludge vermicompost and are often found in the aquatic environment [44,45]. Cryptomycota are mostly endoplasmic parasites of other fungi, such as Chytridiomycetes. Moreover, they regulate the primary production and the debris food chain [45]. A recent study reported that the Cryptomycota (24.92%–31.87%) dominated vermi-wetland with earthworms and plants. Therefore, further studies are needed to assess whether active Cryptomycota dominates sludge vermicompost.

**Figure 5:**
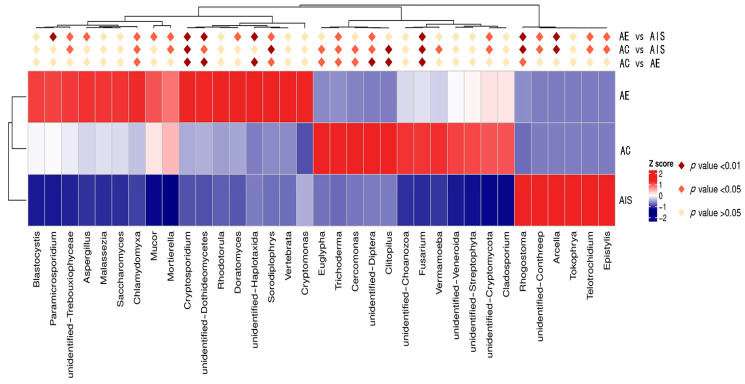
Heatmap of dominant eukaryotic genus and significant differences among initial sludge (AIS) and final products of control (AC) and vermicompost (AE).

The eukaryotic genes of *Rhogostoma*, unidentified *Conthreep*, *Arcella*, *Telotrochidium*, and *Epistylis* were significantly dominant in the initial sludge (Figure 5). Moreover, *Euglypha*, *Trichoderma*, *Cercomonas*, unidentified *Diptera*, *Clitopilus*, and *Fusarium* were significantly dominant in the control (*p* < 0.05). *Chlamydomyxa*, *Cryptosporidium*, unidentified *Dothideomycetes*, unidentified *Haplotaxida*, and *Sorodiplophrys* were significantly dominated in sludge vermicompost (*p* < 0.05). This result also demonstrates that earthworms can insensitively alter the eukaryotic community component in sludge during vermicomposting. The fungal *Chlamydomyxa*, *Cryptosporidium*, and *Dothideomycetes* in sludge vermicompost may suggest they are an important index genus for evaluating maturation degree during sludge vermicomposting. However, *Haplotaxida*, and *Sorodiplophrys,* belonging to the protozoon, dominated in sludge vermicompost, and deserve further exploration.

### 3.4. Correlation Analysis between Environmental Factors and Active Microbes

**Figure 6:**
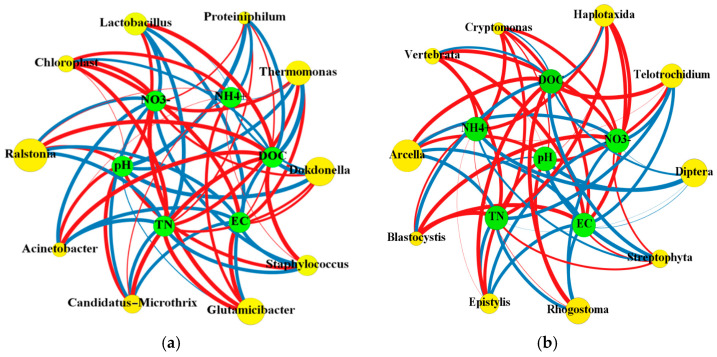
Network diagram showing the relationship between environmental factors and bacterial genus (**a**) or eukaryotic genus; (**b**) dominant in vermicomposting systems. The size of the yellow circle denotes microbial abundance. The thickness of the line indicates the correlation between environmental factors and microorganisms.

Network analysis was used to evaluate the correlation among environmental factors, active bacteria, and eukaryotes. *Dokdonella,* dominant in vermicompost, was negatively correlated with DOC and pH (Figure 6a). However, *Ralstonia* showed an opposite trend. *Glutamicibacter* was positively associated with total nitrogen, nitrate, and EC, while it was negatively associated with DOC. These results indicate that the environmental factors have different effects on the various bacterial genus, similar to the previous study [46]. For the eukaryotic genus, *Arcella*, *Rhogostoma,* and *Telotrochidium* were positively correlated with DOC and pH (Figure 6b). *Haplotaxida*, *Cryptomonas*, *Vertebrata,* and *Epistylis* were significantly positively correlated with ammonium, nitrate, and total nitrogen, indicating that these eukaryotes are involved in nitrogen cycling. These results further suggest that environmental factors can significantly affect different eukaryotic genii.

Canonical correlation analysis can directly reflect the relationship between environmental factors and active microorganisms. Active bacteria were highly correlated with pH and ammonia nitrogen in the environment (Table 2). Dissolved total nitrogen was highly correlated with eukaryotes. The DOC, EC, and nitrate nitrogen were significantly correlated with active bacteria and eukaryotes. The order of correlation between environmental factors and active bacteria was as follows; pH > DOC > nitrate > EC > ammonium > dissolved total nitrogen. The order of correlation between environmental factors and active eukaryotes was as follows; DOC > EC > nitrate > pH > dissolved total nitrogen > ammonium. However, a previous study found that pH has the greatest impact on both bacteria and fungi [47], inconsistent with this study. Therefore, active bacteria and eukaryotes population structure in the vermicomposting system are closely related to environmental factors. The microbial carbon source was significantly correlated with almost all bacteria and eukaryotes compared with the nitrogen source. In addition, previous studies reported that the environmental factors showed a weak relationship with the bacterial community [48], which is also opposite with this study. This is probably associated with the active microbial community (called as functional microbes) detected in this study. Thus, it could be concluded that the earthworms can directly affect the environmental factors and thus modifying the microbial community in vermicomposting.

## 4. Conclusions

Earthworms can decrease the α diversity of active bacteria and eukaryotes, and stimulate the functional microbial community in sludge vermicompost. Proteobacteria and Actinobacteria are the main active bacteria in sludge vermicompost, while Cryptomycota is the dominant active eukaryote phylum. Vermicomposting increases the abundance of bacteria, such as *Glutamicibacter*, *Dokdonella*, *Thermomonas,* and eukaryote *Haplotaxida*. The abundance of active bacteria and eukaryotes is significantly associated with environmental factors in the vermicomposting system, especially the carbon source in sludge. The technology of PMA combined with the DNA sequencing is feasible to detect the functional microorganisms in the environment associated with earthworms.

## Figures and Tables

**Table 1:** Physicochemical properties of raw sludge and final sludge products of vermicompost and control.

Parameters	Initial Sludge	Control	Vermicompost
pH	6.83 ± 0.01 a	6.65 ± 0.04 b	6.76 ± 0.01 c
Electrical conductivity (μS/cm)	200.00 ± 0.20 a	426.33 ± 0.47 b	778.00 ± 1.41 c
Organic matter (%)	37.64 ± 0.38 a	26.67 ± 0.21 b	25.27% ± 0.09 c
Dissolved organic carbon (mg/g)	16.09 ± 0.06 a	3.94 ± 0.06 b	3.53 ± 0.02 c
Ammonium (mg/g)	2.25 ± 0.05	1.95 ± 0.09 a	2.49 ± 0.03 b
Nitrate (mg/g)	0.33 ± 0.01 a	0.90 ± 0.02 b	1.72 ± 0.04 c

Note: The different letters behind the data indicates that the difference is significant between two groups for each parameter.

**Table 2:** Correlation coefficient among bacterial genus, eukaryotic genus, and physicochemical properties of sludge vermicompost.

Parameters	Bacteria	Eukaryotes
pH	0.994 **	0.928
Electrical conductivity	0.976 *	0.98 *
Nitrate	0.984 *	0.976 *
Ammonium	0.969 *	0.876
Total dissolved nitrogen	0.694	0.924
Dissolved organic carbon	0.988 *	0.999 **

Note: * and ** indicate significant level of *p* < 0.05 and *p* < 0.01, respectively.

## Data Availability

No new data were created or analyzed in this study. Data sharing is not applicable to this article.

## References

[1] Dai X.H. (2021). Applications and perspectives of sludge treatment and disposal in China. Science.

[2] Lin L., Li R.H., Li X.Y. (2018). Recovery of organic resources from sewage sludge of Al-enhanced primary sedimentation by alkali pretreatment and acidogenic fermentation. J. Clean. Prod..

[3] Zaharioiu A., Bucura F., Ionete E.I., Ionete R.E., Ebrasu D., Sandru C., Marin F., Oancea S., Niculescu V., Miricioiu M.G. (2020). Thermochemical Decomposition of Sewage Sludge–An Eco-Friendly Solution for a Sustainable Energy Future by Using Wastes. Rev. Chim..

[4] Fu X.Y., Cui G.Y., Huang K., Chen X.M., Li F.S., Zhang X.Y., Li F. (2016). Earthworms facilitate the stabilization of pelletized dewatered sludge through shaping microbial biomass and activity and community. Environ. Sci. Pollut. Res..

[5] Bhat S.A., Singh S., Singh J., Kumar S., Vig A.P. (2018). Bioremediation and detoxification of industrial wastes by earthworms: Vermicompost as powerful crop nutrient in sustainable agriculture. Bioresour. Technol..

[6] Huang K., Xia H., Li F., Bhat S.A. (2020). Recycling of Municipal Sludge by Vermicomposting. Earthworm Assisted Remediation of Effluents and Wastes.

[7] Malafaia G., Costa Estrela D., Guimarães A.T.B., Araújo F.G., Leandro W.M., Lima Rodrigues A.S. (2015). Vermicomposting of different types of tanning sludge (liming and primary) mixed with cattle dung. Ecol. Eng..

[8] Kızılkaya R., Türkay F.Ş.H. (2014). Vermicomposting of Anaerobically Digested Sewage Sludge with Hazelnut Husk and Cow Manure by Earthworm Eisenia foetida. Compost Sci. Util..

[9] Elissen H.J.H., Hendrickx T.L.J., Temmink H., Buisman C.J.N. (2006). A new reactor concept for sludge reduction using aquatic worms. Water Res..

[10] Fu X.Y., Huang K., Cui G.Y., Chen X.M., Li F.S., Zhang X.Y., Li F. (2015). Dynamics of bacterial and eukaryotic community associated with stability during vermicomposting of pelletized dewatered sludge. Int. Biodeterior. Biodegr..

[11] Hu X.R., Zhang T., Tian G.P., Zhang L.M., Bian B. (2021). Pilot-scale vermicomposting of sewage sludge mixed with mature vermicompost using earthworm reactor of frame composite structure. Sci. Total Environ..

[12] Huang K., Xia H., Wu Y., Chen J.Y., Cui G.Y., Li F.S., Chen Y.Z., Wu N. (2018). Effects of earthworms on the fate of tetracycline and fluoroquinolone resistance genes of sewage sludge during vermicomposting. Bioresour. Technol..

[13] Lv B.Y., Xing M.Y., Yang J. (2018). Exploring the effects of earthworms on bacterial profiles during vermicomposting process of sewage sludge and cattle dung with high-throughput sequencing. Environ. Sci. Pollut. Res..

[14] Huang K., Xia H., Zhang Y.Y., Li J.H., Cui G.Y., Li F.S., Bai W., Jiang Y.F., Wu N. (2020). Elimination of antibiotic resistance genes and human pathogenic bacteria by earthworms during vermicomposting of dewatered sludge by metagenomic analysis. Bioresour. Technol..

[15] Domínguez J., Aira M., Gómez-Brandón M., Insam H., Franke-Whittle I., Goberna M. (2010). Vermicomposting: Earthworms enhance the work of microbes. Microbes at Work: From Wastes to Resources.

[16] Aira M., Sampedro L., Monroy F., Domínguez J. (2008). Detritivorous earthworms directly modify the structure, thus altering the functioning of a microdecomposer food web. Soil Biol. Biochem..

[17] Aira M., Monroy F., Domínguez J. (2006). Changes in microbial biomass and microbial activity of pig slurry after the transit through the gut of the earthworm *eudrilus eugeniae* (kinberg, 1867). Biol. Fertil. Soils.

[18] Huang K., Li F.S.F., Wei Y., Chen X.M., Fu X.Y. (2013). Changes of bacterial and fungal community compositions during vermicomposting of vegetable wastes by *Eisenia foetida*. Bioresour. Technol..

[19] Ravindran B., Contreras-Ramos S.M., Sekaran G. (2015). Changes in earthworm gut associated enzymes and microbial diversity on the treatment of fermented tannery waste using epigeic earthworm *Eudrilus eugeniae*. Ecol. Eng..

[20] Chen Y.X., Chang S.K.C., Chen J., Zhang Q., Yu H.Y. (2018). Characterization of microbial community succession during vermicomposting of medicinal herbal residues. Bioresour. Technol..

[21] Blomstrom A.L., Lalander C., Komakech A.J., Vinneras B., Boqvist S. (2016). A metagenomic analysis displays the diverse microbial community of a vermicomposting system in Uganda. Infect. Ecol. Epidemiol..

[22] Nocker A., Camper A.K. (2006). Selective removal of DNA from dead cells of mixed bacterial communities by use of ethidium monoazide. Appl. Environ. Microbiol..

[23] Nocker A., Sossa-Fernandez P., Burr M.D., Camper A.K. (2007). Use of Propidium Monoazide for Live/Dead Distinction in Microbial Ecology. Appl. Environ. Microbiol..

[24] Van Frankenhuyzen J.K., Trevors J.T., Lee H., Flemming C.A., Habash M.B. (2011). Molecular pathogen detection in biosolids with a focus on quantitative PCR using propidium monoazide for viable cell enumeration. J. Microbiol. Method.

[25] VSEARCH Binaries. https://github.com/torognes/vsearch/.

[26] Silva132 Database. http://www.arb-silva.de/.

[27] Wu Z., Yin B., Song X., Qiu J., Cao L., Zhao Q. (2019). Effects of salinity on earthworms and the product during vermicomposting of kitchen wastes. Int. J. Environ. Res. Public Health.

[28] Xia H., Wu Y., Chen X.M., Huang K., Chen J.Y. (2019). Effects of antibiotic residuals in dewatered sludge on the behavior of ammonia oxidizers during vermicomposting maturation process. Chemosphere.

[29] Lv B., Zhang D., Chen Q., Cui Y. (2019). Effects of earthworms on nitrogen transformation and the correspond genes (amoA and nirS) in vermicomposting of sewage sludge and rice straw. Bioresour. Technol..

[30] Hartenstein R., Hartenstein F. (1981). Physicochemical changes effected in activated sludge by the earthworm *Eisenia foetidal*. J. Environ. Qual..

[31] Zhao C.H., Wang Y., Wang Y., Wu F.J., Zhang J.G., Cui R.Y., Wang L.G., Mu H. (2018). Insights into the role of earthworms on the optimization of microbial community structure during vermicomposting of sewage sludge by PLFA analysis. Waste Manag..

[32] Drake H.L., Horn M.A. (2007). As the worm turns: The earthworm gut as a transient habitat for soil microbial biomes. Annu. Rev. Microbiol..

[33] Aira M., Olcina J., Pérez-Losada M., Domínguez J. (2016). Characterization of the bacterial communities of casts from Eisenia andrei fed with different substrates. Appl. Soil Ecol..

[34] Bonkowski M., Schaefer M. (1997). Interactions between earthworms and soil protozoa: A trophic component in the soil food web. Soil Biol. Biochem..

[35] Monroy F., Aira M., Domínguez J. (2008). Changes in density of nematodes, protozoa and total coliforms after transit through the gut of four epigeic earthworms (oligochaeta). Appl. Soil Ecol..

[36] Xia Y., Wen X.H., Zhang B., Yang Y.F. (2018). Diversity and assembly patterns of activated sludge microbial communities: A review. Biotechnol. Adv..

[37] Wang Y., Han W., Wang X.Y., Chen H.M., Zhu F., Wang X.P., Lei C.L. (2017). Speciation of heavy metals and bacteria in cow dung after vermicomposting by the earthworm, *Eisenia Fetida*. Bioresour. Technol..

[38] Domínguez J., Aira M., Kolbe A.R., Gómez-Brandón M., Pérez-Losada M. (2019). Changes in the composition and function of bacterial communities during vermicomposting may explain beneficial properties of vermicompost. Sci. Rep..

[39] Borker S.S., Thakur A., Kumar S., Kumari S., Kumar R., Kumar S. (2021). Comparative genomics and physiological investigation supported safety, cold adaptation, efficient hydrolytic and plant growth-promoting potential of psychrotrophic *Glutamicibacter arilaitensis* LJH19, isolated from night-soil compost. BMC Genom..

[40] Hyun D.W., Sung H., Kim P.S., Yun J.H., Bae J.W. (2021). Leucobacter coleopterorum sp. nov., Leucobacter insecticola sp. nov., and Leucobacter viscericola sp. nov., isolated from the intestine of the diving beetles, Cybister brevis and Cybister lewisianus, and emended description of the genus Leucobacter. J. Microbiol..

[41] Matsunaga K., Kubota K., Harada H. (2014). Molecular diversity of eukaryotes in municipal wastewater treatment processes as revealed by 18s rRNA gene analysis. Microbes. Environ..

[42] Sapkota R., Santos S., Farias P., Krogh P.H., Winding A. (2020). Insights into the earthworm gut multi-kingdom microbial communities. Sci. Total Environ..

[43] Monroy F., Aira M., Domínguez J. (2011). Epigeic earthworms increase soil arthropod populations during first steps of decomposition of organic matter. Pedobiologia.

[44] Chouari R., Leonard M., Bouali M., Guermazi S., Rahli N., Zrafi I., Morin L., Sghir A. (2017). Eukaryotic molecular diversity at different steps of the wastewater treatment plant process reveals more phylogenetic novel lineages. World J. Microbiol. Biotechnol..

[45] Gleason F.H., Carney L.T., Lilje O., Glockling S.L. (2012). Ecological potentials of species of rozella (Cryptomycota). Fungal Ecol..

[46] Zhang X.W., Song Z.J., Tang Q.D., Wu M.H., Zhou H., Liu L.F., Qu Y.U. (2021). Performance and microbial community analysis of bioaugmented activated sludge for nitrogen-containing organic pollutants removal. J. Environ. Sci..

[47] Cai H.B., Feng W.W., Dong Y.H., Ma Z.L., Cao H.J., Sun J.D., Zhang B.G. (2020). Microbial community succession in industrial composting with livestock manure and peach branches and relations with environmental factors. Environ. Sci..

[48] Xia H., Huang K. (2021). Effects of TiO_2_ and ZnO nanoparticles on vermicomposting of dewatered sludge: Studies based on the humification and microbial profiles of vermicompost. Environ. Sci. Pollut. Res..

